# Metabolic versatility of aerobic methane‐oxidizing bacteria under anoxia in aquatic ecosystems

**DOI:** 10.1111/1758-2229.70002

**Published:** 2024-09-04

**Authors:** Biao Li, Zhendu Mao, Jingya Xue, Peng Xing, Qinglong L. Wu

**Affiliations:** ^1^ Key Laboratory of Lake and Watershed Science for Water Security, Nanjing Institute of Geography and Limnology Chinese Academy of Sciences Nanjing China; ^2^ State Key Laboratory of Lake Science and Environment, Nanjing Institute of Geography and Limnology Chinese Academy of Sciences Nanjing China; ^3^ Center for Evolution and Conservation Biology Southern Marine Science and Engineering Guangdong Laboratory (Guangzhou) Guangzhou China; ^4^ School of Geographical Sciences Nanjing Normal University Nanjing China; ^5^ Sino‐Danish Center for Education and Research University of Chinese Academy of Sciences Beijing China; ^6^ The Fuxianhu Station of Plateau Deep Lake Research Chinese Academy of Sciences Yuxi China

## Abstract

The potential positive feedback between global aquatic deoxygenation and methane (CH_4_) emission emphasizes the importance of understanding CH_4_ cycling under O_2_‐limited conditions. Increasing observations for aerobic CH_4_‐oxidizing bacteria (MOB) under anoxia have updated the prevailing paradigm that MOB are O_2_‐dependent; thus, clarification on the metabolic mechanisms of MOB under anoxia is critical and timely. Here, we mapped the global distribution of MOB under anoxic aquatic zones and summarized four underlying metabolic strategies for MOB under anoxia: (a) forming a consortium with oxygenic microorganisms; (b) self‐generation/storage of O_2_ by MOB; (c) forming a consortium with non‐oxygenic heterotrophic bacteria that use other electron acceptors; and (d) utilizing alternative electron acceptors other than O_2_. Finally, we proposed directions for future research. This study calls for improved understanding of MOB under anoxia, and underscores the importance of this overlooked CH_4_ sink amidst global aquatic deoxygenation.

## Graphical abstract

Besides conventional aerobic oxidation pathway, MOB has four underlying metabolic strategies under anoxia: (i) forming a consortium with oxygenic microorganisms; (ii) self‐generation/storage of O_2_ by MOB; (iii) forming a consortium with non‐oxygenic heterotrophic bacteria that use other electron acceptors; (iv) and utilizing alternative electron acceptors other than O_2_, such as NO_X_^−^, N_2_O, and Fe(III).

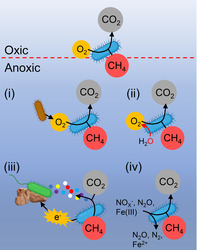

## INTRODUCTION

Dissolved oxygen (DO) in aquatic ecosystems has declined over the past half‐century, primarily because of global warming (Breitburg et al., 2018; Jane et al., 2021; Schmidtko et al., 2017; Zhi et al., 2023). Besides reducing the solubility of O_2_, greenhouse gas‐driven warming raises metabolic rates and results in accelerating aquatic O_2_ consumption (Keeling et al., 2010). Meanwhile, warming‐induced intensified stratification accounts for significant O_2_ loss by impeding ventilation, hindering O_2_ transport from the surface to deeper layers (Helm et al., 2011). Moreover, once exposed to hypoxic or anoxic conditions due to deoxygenation, methanogens potentially activate the production of CH_4_, a greenhouse gas which is 28 times more potent in holding heat than carbon dioxide (CO_2_) on a centennial timescale (Tollefson, 2022). This means that deoxygenation is likely to exert positive feedback on CH_4_ emission (Bonaglia et al., 2022; Chronopoulou et al., 2017). Given that aquatic ecosystems contribute nearly half of global CH_4_ emission (Rosentreter et al., 2021), even slight deoxygenation may trigger serious ecological consequences.

CH_4_ emission is a balance between production and oxidation (He et al., 2018; Zhu et al., 2020). Depending on whether the electron acceptor is O_2_, CH_4_ oxidation can be divided into aerobic and anaerobic CH_4_ oxidation. Since O_2_ is a thermodynamically favourable electron acceptor for CH_4_ oxidation, aerobic CH_4_‐oxidizing bacteria (MOB), a group of bacteria that grow on CH_4_ as their sole source of carbon and energy (Kalyuzhnaya et al., 2019), are considered a critical biofilter to mitigate CH_4_ emission (Mao et al., 2022). In the absence of O_2_, anaerobic CH_4_‐oxidizing archaea (ANME) consume CH_4_ via a reverse methanogenic pathway coupled with other electron acceptors like sulfate (SO_4_^2−^), nitrate (NO_3_^−^), and metal oxides (Boetius et al., 2000; Raghoebarsing et al., 2006; Beal et al., 2009; Haroon et al., 2013), and the NC10‐bacteria related to *Candidatus Methylomirabilis oxyfera* (*M*. *oxyfera*) produce O_2_ intracellularly from nitrite (NO_2_^−^) for CH_4_ oxidation (Ettwig et al., 2010). The established norm in microbial ecology is that CH_4_ metabolisms by MOB are O_2_‐dependent; thus, MOB are traditionally believed to thrive only in oxic environments, especially at the oxic–anoxic interface where diffusion of CH_4_ from below and DO from above provide a suitable niche for them (Reim et al., 2012). Nevertheless, increasing studies have demonstrated that MOB can survive and even actively metabolize CH_4_ in environments with very low or even undetectable O_2_ concentrations (Figure S1). These unexpected findings have substantially updated the understanding that the ecological amplitude of MOB is broader than previously recognized, and that the role of MOB in mitigating global warming under anoxia may be neglected (Reis et al., 2024). However, our understanding of the metabolic strategies employed by MOB under anoxia remains limited.

To fill the knowledge gap, we mapped the presence of MOB in global anoxic environments, summarized four metabolic strategies for MOB survival under anoxia, and proposed directions for future research.

## UBIQUITOUS PRESENCE OF MOB IN ANOXIC ENVIRONMENTS

**FIGURE 1:**
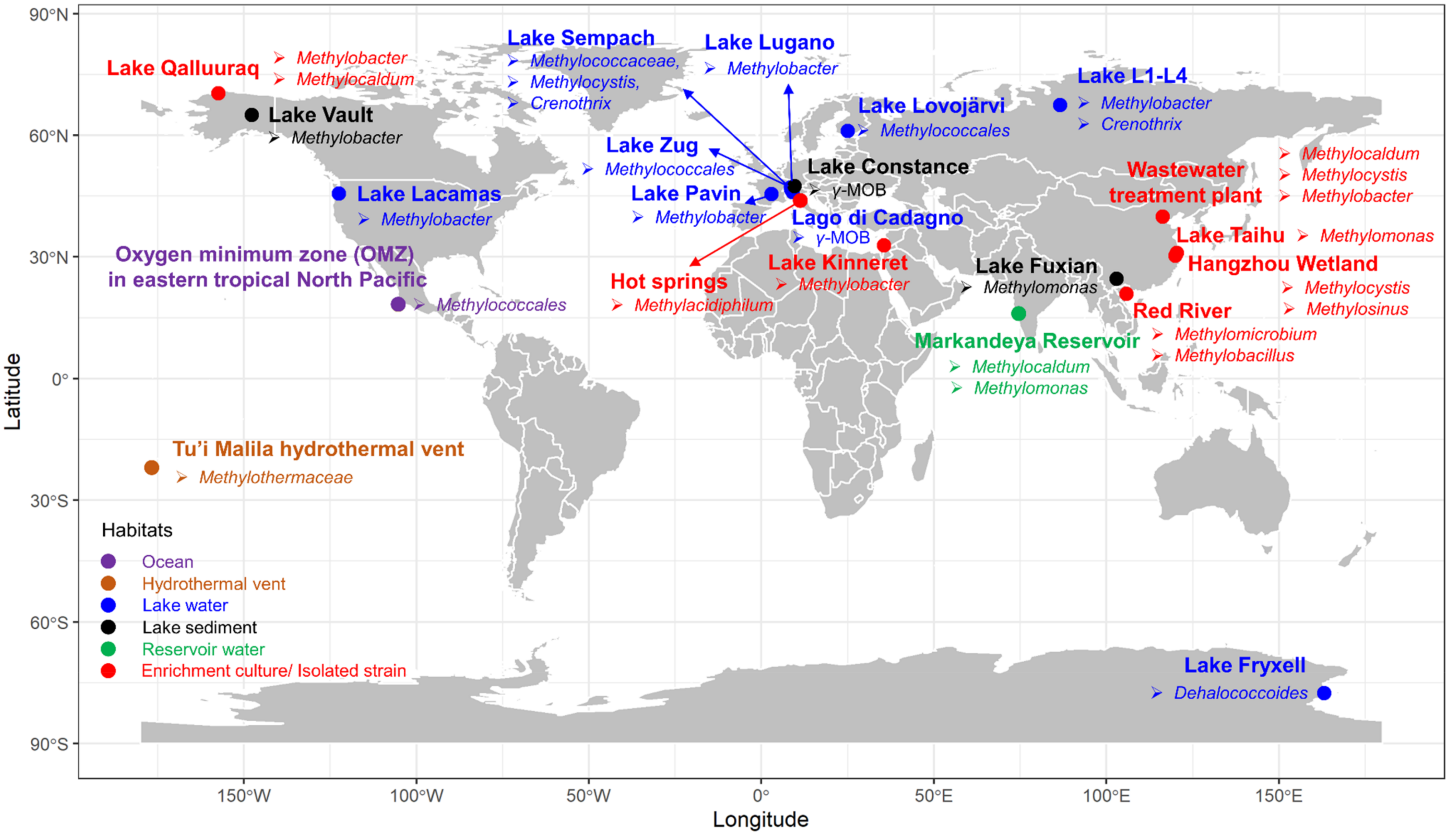
Global distribution of MOB under anoxia in typical aquatic ecosystems. The purple, brown, blue, black, green, and red filled circle represent observations from the ocean, hydrothermal vent, lake water, lake sediment, reservoir, and enrichment culture or isolated strain from the above habitats respectively.

An absolute anoxic environment (i.e., zero O_2_ concentration) cannot be directly detected because no assay has detection limit low enough, typically reaching only nanomolar level (Berg et al., 2022). However, many bacteria can sense and utilize O_2_ in nanomolar concentrations that escape most detection attempts, making a vague boundary between aerobes and anaerobes (Bristow et al., 2016; Kalvelage et al., 2015; Stolper et al., 2010; Trojan et al., 2021). This capability among traditionally considered aerobes indicates that they play a broader environmental role and possess more versatile metabolic pathways than those currently recognized (Berg et al., 2022; Trojan et al., 2021). Here, we operationally define the “apparent anoxic” conditions as those where DO levels drop below the detection limit of O_2_ sensing technologies (Canfield & Kraft, 2022). Recent studies have identified MOB in anoxic environments globally, especially in aquatic ecosystems including oceans, hydrothermal vents, lakes, and reservoirs (Figure 1 and Table 1). Some cultures and strains enriched or isolated from these habitats also have shown that MOB actively consume CH_4_ under O_2_‐limited or depleted conditions (Figure 1 and Table 1). Intriguingly, several studies have identified MOB as the sole CH_4_ consumer with no detection of anaerobic methanotrophs (e.g., ANME‐type archaea and NC10‐bacteria) (Dershwitz et al., 2021; Milucka et al., 2015). Moreover, Gammaproteobacterial‐MOB, such as *Methylobacter* and *Methylomonas*, are frequently found in anoxic environments (He et al., 2022; Li et al., 2023; Thamdrup et al., 2019). These MOB under anoxia play an unexpected important role in mitigating CH_4_ emission. For instance, MOB were identified as responsible for 40.3% of CH_4_ reduction in the anoxic sediments of Lake Fuxian (Li et al., 2023), and nearly complete consumption of CH_4_ in the anoxic waters of Lake Lago di Cadagno (Milucka et al., 2015). Although these types of MOB sometimes catalyse CH_4_ under anoxia non‐syntrophically (e.g., Kits et al., 2015), in more cases they appear to aggregate with other microorganisms (e.g., Shi et al., 2021), indicating elaborate metabolic mechanisms are involved.

**TABLE 1:** Aerobic methane‐oxidizing bacteria (MOB) under anoxia in typical aquatic ecosystems and laboratory systems.

Habitats	Depth (W/S)^a^	Temp (°C)	O_2_ detection limits (μmol/L)	Key MOB taxa	Electron acceptors	Metabolic pathways^b^	Accession number^c^	References
Lake Constance	S: 0.5–9	‐	0.3	γ‐MOB	‐	‐	‐	Rahalkar et al. (2009)
Lake Pavin	W: 65	4	1	*Methylobacter*	‐	‐	‐	Biderre‐Petit et al. (2011)
Lake Lugano	W: 135–220	‐	1	*Methylobacter*	‐	‐	‐	Blees et al. (2014)
Strain obtained from Jay Gulledge	‐	30	0.05	*Methylomonas*	NO_3_^−^	c/d	PRJNA258394	Kits et al. (2015)
Lake Lago di Cadagno	W: 13	‐	1	γ‐MOB	O_2_	a	‐	Milucka et al. (2015)
Tu'i Malila hydrothermal vent	W: 1876	10–12	‐	*Methylothermaceae*	NO_3_^−^	c/d	IMG2623620619	Skennerton et al. (2015)
Lake Zug	W: 180–190	‐	0.02	*Methylococcales*	O_2_, NO_3_^−^, NO_2_^−^, Fe(III), Mn(IV)	‐	PRJNA977988	Oswald et al. (2016) and Schorn et al. (2024)
Lake Fryxell	S: 10	2.1–2.4	‐	*Dehalococcoides*	Humic acids	‐	‐	Saxton et al. (2016)
Lake Kinneret	S: 26–41	20	0.03125	*Methylobacter*	Fe(III)	c	‐	Bar‐Or et al. (2017)
Lake Vault	S: 0–2.5	‐	‐	*Methylobacter*	Fe(III), Mn(IV)	‐	‐	Martinez‐Cruz et al. (2017)
Markandeya Reservoir	W: 12–22	23	2^d^	*Methylocaldum*, *Methylomonas*	NO_3_^−^, NO_2_^−^	a/c/d	‐	Naqvi et al. (2018)
Eastern tropical North Pacific	W: 100–400	14	0.001–0.01^e^	*Methylcoccales*	‐	‐	PRJNA263621	Thamdrup et al. (2019)
Lake L1‐L4	W: 7–9.6	3.4–3.9	0.3125	*Methylobacter*, *Crenothrix*	NO_3_^−^, NO_2_^−^	a/c/d	‐	Cabrol et al. (2020)
Enrichment from wastewater treatment plant	‐	‐	‐	*Methylocaldum*, *Methylocystis*, *Methylobacter*	O_2_	a	‐	Chai et al. (2020)
Lake Lacamas	W: 5–18	10–20	‐	*Methylobacter*	NO_3_^−^	c	PRJNA524776	van Grinsven et al. (2020, 2021)
Enrichment from Lake Taihu	‐	30	‐	*Methylomonas*	O_2_	a	‐	Chang et al. (2021)
Stains purified from the spent medium	‐	25	‐	*Methylocystis*, *Methylosinus*	O_2_	b	‐	Dershwitz et al. (2021)
A meander bend of the Red River	S: 3000	26	‐	*Methylomicrobium*, *Methylobacillus*	‐	a/c/d	‐	Pienkowska et al. (2021)
Lake Lovojärvi	W: 11	7.5	1	*Methylococcales*	NO_3_^−^, NO_2_^−^	d	PRJEB38681	Rissanen et al. (2021)
Enrichment from wetland sediment in Hangzhou	‐	‐	‐	*Methylocystis*, *Methylosinus*	SeO_4_^2−^	c	SRP136677, SRP136696, SRP136790, SRP136859	Shi et al. (2021)
Lake Qalluuraq	S: 25–50	10	‐	*Methylobacter*, *Methylocaldum*	Fe(III)	c	MN788533‐MN788604, SRP234857	He et al. (2022)
Lake Sempach	S: 0–10	‐	‐	*Methylococcaceae*, *Methylocystis*, *Crenothrix*	O_2_	a/b	‐	Su et al. (2022)
Lake Fuxian	S: 0–10	13	0.625	*Methylomonas*	Fe(III)	c	OEP002958	Li et al. (2023)
Strains isolated from hot spring in Italy	‐	‐	‐	*Methylacidiphilum*	N_2_O	d	CP065957	Awala et al. (2024)

^a^ Depth (W/S): depth for MOB present in the anoxic zone of water column (W) or sediment (S), with unit of m and cm, respectively.

^b^ Metabolic pathways: (a) forming a consortium with oxygenic microorganisms; (b) self‐generation/ storage of O_2_ by MOB; (c) forming a consortium with non‐oxygenic heterotrophic bacteria that use other electron acceptors; (d) utilizing alternative electron acceptors other than O_2_.

^c^ Accession number is only for (meta)genomic sequencing data but not for amplicon sequencing data.

^d^ O_2_ detection limit from Labasque et al. (2004).

^e^ O_2_ detection limit from Revsbech et al. (2009).

## METABOLIC VERSATILITY OF MOB UNDER ANOXIA

**FIGURE 2:**
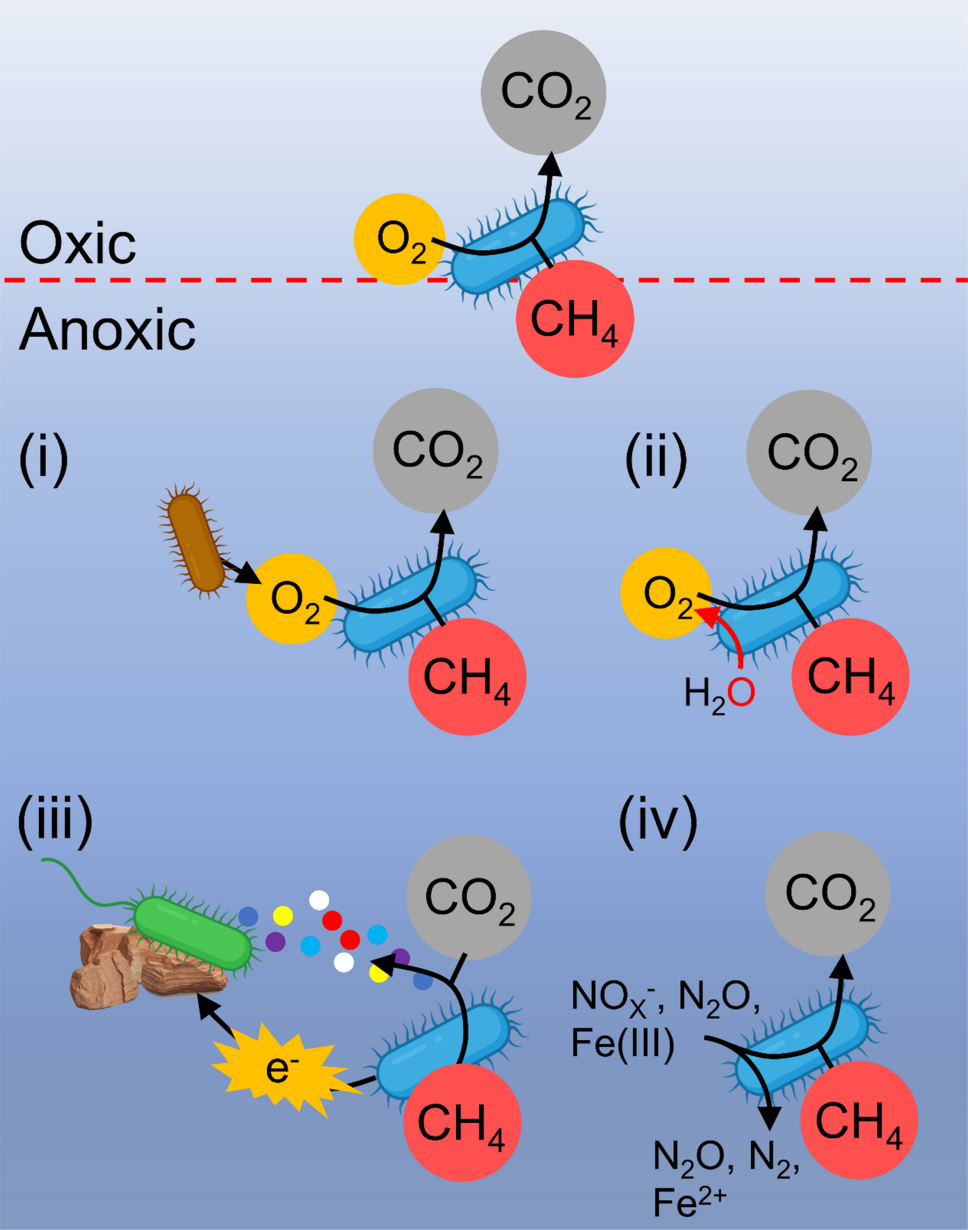
Conventional aerobic metabolic pathway in oxic environments and four potential metabolic strategies under anoxia for MOB: (i) forming a consortium with oxygenic organisms; (ii) self‐generation/storage of O_2_ by MOB; (iii) forming a consortium with non‐oxygenic heterotrophic bacteria that use other electron acceptors; (iv) utilizing alternative electron acceptors other than O_2_, such as NO_X_^−^, N_2_O, and Fe(III).

The conventional aerobic oxidation pathway of MOB has been extensively reviewed in previous studies (Hanson & Hanson, 1996; Kalyuzhnaya et al., 2019). Briefly, initiated by CH_4_ monooxygenase (MMO) to split the O—O bonds under the presence of O_2_, MOB activate and oxidize CH_4_ to methanol (Hanson & Hanson, 1996; Murrell et al., 2000), followed by oxidation to formaldehyde by methanol dehydrogenase (MDH) with a subunit of MxaF or XoxF (Chistoserdova, 2016; McDonald & Murrell, 1997). Formaldehyde oxidation can be catalysed by several enzymes, including the tetrahydromethanopterin‐ (H_4_MPT‐) or tetrahydrofolate linked and formaldehyde dehydrogenases (FADH, possibly also XoxF) (Kalyuzhnaya et al., 2019). Besides being oxidized via formate to CO_2_ by FADH and formate dehydrogenase (FDH), another part of formaldehyde is assimilated into the biomass via the Serine pathway or the ribulose monophosphate (RuMP) pathway (Chistoserdova et al., 2005). However, MMO inhibition under anoxia suppresses the initial oxidation step in CH_4_ transformation to methanol (Roslev & King, 1995). Therefore, MOB require distinct strategies to use CH_4_ as a carbon and energy source under anoxia, as summarized below (Figure 2).

### Forming a consortium with oxygenic microorganisms (Figure 2i)

**FIGURE 3:**
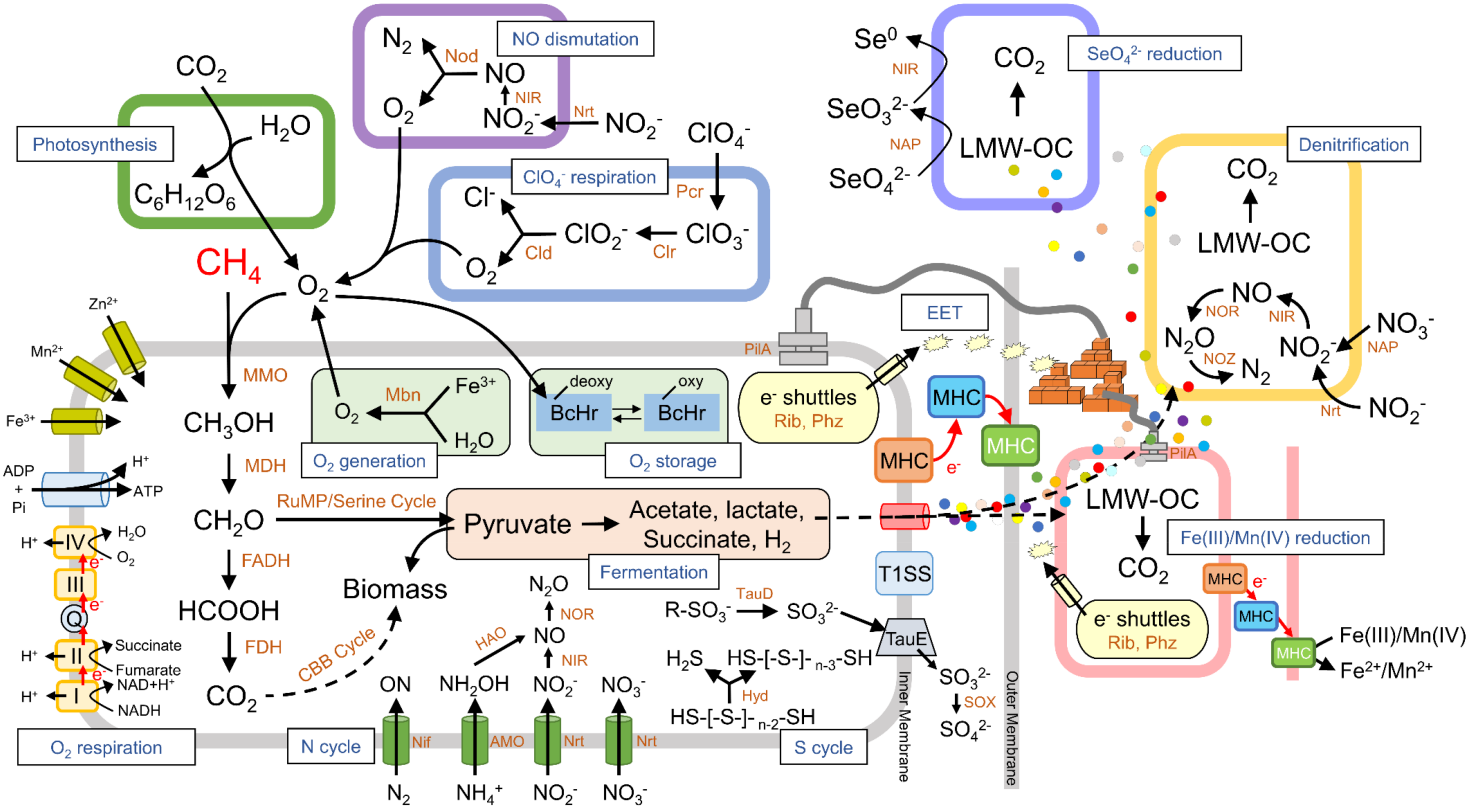
Metabolic pathway for MOB under anoxia. CH_4_ oxidation module: MMO, methane monooxygenase; MDH, methanol dehydrogenase; FADH, formaldehyde dehydrogenase; FDH, formate dehydrogenase; LMW‐OC, low molecular weight organic carbon. O_2_ generation/storage module: Mbn, methanobactin synthetase; BcHr, bacteriohemerythrin. EET (extracellular electron transfer) module: Rib, riboflavin synthetase; Phz, phenazine synthetase; PilA, the key protein to constitute electrically conductive pili (e‐pili); MHC, putative multiheme *c*‐type cytochromes; T1SS, Type 1 secretion system. Nitrogen cycle module: ON, Organic nitrogen; Nif, nitrogenase; AMO, ammonia monooxygenase; HAO, hydroxylamine dehydrogenase; NAP, nitrate reductase; Nrt, nitrate/nitrite transporter; NIR, nitrite reductase; NOR, nitric oxide reductase; NOD, nitric oxide dismutase; NOZ, nitrous oxide reductase. Sulfur cycle module: TauD, taurine dioxygenase; Hyd, sulfhydrogenase; SOX, sulfite oxidase. ClO_4_^−^ respiration module: Pcr, perchlorate reductase; Clr, chlorate reductase; Cld, chlorite dismutase.

Coupling CH_4_ oxidation with O_2_ respiration yields more energy for MOB relative to other electron acceptors except for NO_2_^−^ (Table S1). Since only few MOB types possess key genes encoding NO_x_^−^ reduction (Kits et al., 2015; Rissanen et al., 2021) and O_2_ is toxic to NO_x_^−^ reducers (Lu & Imlay, 2021), MOB will be thermodynamically favourable to utilize O_2_ as the electron acceptors when trace O_2_ is simultaneously present with other electron acceptors. Consequently, MOB are frequently found to aggregate with oxygenic organisms under anoxia (Milucka et al., 2015; Oswald et al., 2015; Thamdrup et al., 2019). To date, only few biological pathways are known to produce O_2_ (Ettwig et al., 2012; Kraft et al., 2022): photosynthesis (Dismukes et al., 2001), nitric oxide (NO) dismutation (Ettwig et al., 2010), chlorate respiration (van Ginkel et al., 1996), detoxification of reactive oxygen species (ROS) (Apel & Hirt, 2004), and a new pathway by ammonia‐oxidizing archaea (AOA) which has not been completely resolved (Kraft et al., 2022). The O_2_ produced by these oxygenic pathways can be immediately consumed by aerobic microorganisms (including MOB) through a conventional aerobic respiration, making O_2_ undetectable (Figure 3, Milucka et al., 2015).

In the permanently stratified Lake Lago di Cadagno, where the euphotic layer extends below the oxic–anoxic interface, abundant MOB affiliated to Gammaproteobacteria were attached to photosynthetic algae and actively consume CH_4_ in the anoxic water column (Milucka et al., 2015). Likewise, *Methylomonas* was also enriched alongside the oxygenic NC10‐bacteria in a NO_2_^−^‐dependent anaerobic CH_4_ oxidation system, indicating that *Methylomonas* was fuelled by O_2_ through NO_2_^−^‐derived NO dismutation pathway (Chang et al., 2021). A recent study found abundant and diverse nitric oxide dismutase (NOD) genes affiliated not only with NC10‐bacteria but also with diverse heterotrophic bacteria, such as *Flavihumibacter*, *Pseudomonas* and a new order (UBA11136) of Alphaproteobacteria (Elbon et al., 2024; Zhu et al., 2017). This implies that different combinations between MOB and oxygenic bacteria may widely occur in anoxic environments. Additionally, in a CH_4_‐based membrane biofilm batch reactor that used perchlorate (ClO_4_^−^) as the electron acceptor for anaerobic CH_4_ oxidation, multiple MOB genera, including *Methylococcus*, *Methylomonas*, and *Methylocystis* accounted for 20%–27% of the total bacteria and were likely involved in CH_4_ oxidation (Lv et al., 2019). Since perchlorate‐reducing bacteria reduce ClO_4_^−^ to chlorite (ClO_2_^−^), which is further intracellularly disproportionated to chloride (Cl^−^) and O_2_ (Miller et al., 2014), MOB could use O_2_ produced by perchlorate respiration to oxidize CH_4_ and instant O_2_ recycling would maintain anoxic conditions. Because numerous heterotrophic bacteria have a capacity to produce ROS (Diaz et al., 2013), which can be detoxified to O_2_ by superoxide dismutase (SOD) and catalase (CAT) with hydrogen peroxide (H_2_O_2_) as the intermediate product (Apel & Hirt, 2004), it is speculated that MOB can use ROS‐detoxified O_2_ to oxidize CH_4_ under anoxia. However, to the best of our knowledge, the detoxification of ROS coupled with CH_4_ oxidation mediated by MOB has not been reported up to now.

### Self‐generation/storage of O_2_ by MOB (Figure 2ii)

Self‐generation of O_2_ by MOB is dependent on the extracellular copper‐binding peptides called methanobactins (MBs, Figure 3) (Dershwitz et al., 2021). Based on MBs, some Alphaproteobacterial‐MOB have novel acquisition systems for copper ions (Cu^2+^) (DiSpirito et al., 2016), that are critical for regulating MMO expression (Murrell et al., 2000). Intriguingly, MBs can bind with multiple metal ions besides Cu^2+^ and reduce some bound ions (Choi et al., 2006; Lu et al., 2017). By using an H_2_^18^O tracing method, two Alphaproteobacterial‐MOB strains, *Methylosinus trichosporium* OB3b and *Methylocystis* sp. strain SB2, were found to produce ^36^O_2_ when incubated in the presence of either Au^3+^, Cu^2+^, or Ag^+^ (Dershwitz et al., 2021). Furthermore, ^36^O_2_ was generated by coupling Fe^3+^ reduction and H_2_^18^O oxidation with the help of MBs (Dershwitz et al., 2021). This is the first evidence verifying “self‐generation” of O_2_ by MOB and the ability to express MBs (thereby generate O_2_) may be an important pathway for facilitating CH_4_ removal under anoxia. Notably, most MOB found in anoxic zones are affiliated to Gammaproteobacteria (Figure 1 and Table 1). Although some Gammaproteobacterial‐MOB can secrete copper‐binding compounds (Choi et al., 2010), none of these have been verified to have genes encoding MBs biosynthesis as of yet (Semrau et al., 2020). Therefore, it is possible that Gammaproteobacterial‐MOB generate O_2_ via some unknown mechanism or utilize O_2_ produced by others through MBs production. Similarly, AOA, which have long been considered O_2_‐dependent, were also recently found to self‐produce O_2_ (Kraft et al., 2022). These studies indicate that “self O_2_ generation” maybe an overlooked capacity for conventional aerobic microorganisms surviving anoxic environments.

Storage of O_2_ by MOB mainly relies on the O_2_‐carrier bacteriohemerythrin (Figure 3), which shuttles O_2_ from the cytoplasm of the cell to the intra‐cytoplasmic membranes for consumption by particulate MMO (Chen et al., 2012). Under O_2_‐limited conditions, one notable change in the transcriptomes of MOB, such as *Methylomonas denitrificans* FJG1, *Methylococcus capsulatus* (Bath), and *Methylomicrobium buryatense* 5GB1C, is the upregulation of genes encoding bacteriohemerythrin (Chen et al., 2012; Gilman et al., 2017; Kits et al., 2015). This means that O_2_ from episodically inputs like turbidity currents from surface water into the anoxic zone, is likely stored or consumed quickly instead of being detected during sampling campaigns (Blees et al., 2014). However, further field evidences are required to reveal the importance of O_2_ storage by MOB for CH_4_ removal under anoxic conditions.

### Forming a consortium with non‐oxygenic heterotrophic bacteria that use other electron acceptors (Figure 2iii)

MOB can transform CH_4_ to low molecular weight compounds via the pyrophosphate‐mediated glycolytic pathway under micro‐oxic conditions (Kalyuzhnaya et al., 2013; Khanongnuch et al., 2023). These compounds can be utilized as carbon sources by heterotrophic bacteria, establishing a syntrophic link between methanotrophy and heterotrophy (He et al., 2015). Moreover, trace O_2_ seems to only trigger the reaction instead of acting as an electron acceptor for CH_4_ oxidation, because the amount of O_2_ consumption is much lower than that of CH_4_ oxidation. In a membrane biofilm reactor, MOB transformed CH_4_ to methanol and excreted part of it out of the cells, which was further assimilated by methanol‐utilizing denitrifiers at a low O_2_:CH_4_ ratio (0.06, Xu et al., 2020).

Syntrophy is not limited to micro‐oxic environments. Even under anoxic conditions, MOB can still form a consortium with heterotrophic bacteria. Some field investigations found that MOB may couple CH_4_ oxidation with NO_x_^−^ reduction in the anoxic water columns of freshwater lakes (Rissanen et al., 2018; van Grinsven et al., 2021). An in situ observation also revealed the co‐existence of MOB and iron reducers below the sulfate–methane transition zone (SMTZ) in the sediment of a boreal estuary, indicating a potential linkage between them by using Fe(III) as an alternative electron acceptors (Myllykangas et al., 2020). When solid electron acceptors such as Fe(III) oxides dominate anoxic environments, extracellular electron transfer (EET), including putative multiheme *c*‐type cytochromes (MHCs), electrically conductive pili (e‐pili), and electron shuttles (such as flavins, phenazine, and rebredoxin), is likely to play a critical role (Shi et al., 2016). Our recent study showed that the *pilA* gene encoding e‐pili, the genes encoding putative and extracellular periplasmic MHCs, and the genes encoding electron shuttles particularly riboflavin, including *ribA*, *ribBA*, *ribD*, *ribE*, *ribF*, and *ribH*, were all present in *Methylomonas* (Figure 3, Li et al., 2023). Furthermore, MOB form a consortium with some heterotrophic bacteria to utilize ferrihydrite as an alternative electron acceptor under anoxia with the help of riboflavin (Li et al., 2023). Given the ubiquitous presence of electron shuttles and low molecular weight compounds in natural anoxic sediments, it is possible that similar MOB consortia exist and function in situ. This has been further verified in Arctic lake sediments, where MOB actively oxidize ^13^CH_4_ and generate intermediates like methanol, formaldehyde, and formate, which fuel ferric reduction via dissimilatory iron‐reducing bacteria (He et al., 2022). In an anoxic membrane biofilm batch reactor, MOB excrete some fermentation by‐products including formate, acetate, propionate, butyrate, and lactate for heterotrophic bacteria (such as *Pseudoxanthomonas*, *Piscinibacter*, and *Rhodocyclaceae*), and the latter reduce selenate to selenite and elemental selenium by proteins annotated as periplasmic NO_3_^−^ reductases (Shi et al., 2021).

### Utilizing alternative electron acceptors other than O_2_ (Figure 2iv)

Previous studies have shown that anaerobic methanotrophs can link CH_4_ oxidation to the reduction of alternative electron acceptors under anoxia (Cai et al., 2021; Oni & Friedrich, 2017). In addition to the conventional O_2_‐dependent pathway for CH_4_ oxidation, some MOB have the potential to use alternative electron acceptors such as NO_x_^−^ and Fe(III) under anoxia when oxygenic organisms are absent (Kits et al., 2015; Zheng et al., 2020). It has been reported that a Gammaproteobacterial‐MOB *Methylomonas denitrificans* sp. nov. strain FJG1^T^, couples CH_4_ oxidation to NO_3_^−^ reduction when DO was undetectable, releasing N_2_O as a terminal product (Kits et al., 2015). Transcriptomic analysis further revealed the upregulation of genes encoding the denitrification pathway upon NO_3_^−^ amendment under anoxia within this MOB strain (Kits et al., 2015). Some acidophilic Alphaproteobacterial‐MOB strains, such as *Methylocella tundrae* T4 and *Methylacidiphilum caldifontis* IT6, possess N_2_O reductase genes and were recently shown to consume CH_4_ under anoxia using N_2_O as the terminal electron acceptor (Awala et al., 2024). However, the genetic potential for N_2_O respiration by Gammaproteobacterial‐MOB, which are ubiquitously distributed in anoxic aquatic systems, remains constrained. Besides coupling CH_4_ oxidation and NO_3_^−^/N_2_O reduction, key genes encoding N_2_ fixation (*nifDHK*) were present within the genome of MOB in freshwater lakes and oxygen minimum zones of the oceans (Figure 3, Jayakumar & Ward, 2020; Rissanen et al., 2021; Khanongnuch et al., 2022), indicating that MOB also have genetic potential for nitrogen fixation under O_2_‐limited environments (Murrell & Dalton, 1983). Additionally, MOB strains belonging to Alpha‐ and Gammaproteobacteria, *Methylosinus* sp. LW4 and *Methylomonas* sp. LW13, were found to couple ferrihydrite reduction with CH_4_ oxidation under O_2_ limitation (initial DO of 0.89 mg/L). Although genes encoding outer membrane cytochromes were absent within *Methylomonas* sp. LW13, the expression of one conspicuous gene cluster encoding the Type 1 secretion system (T1SSs) was upregulated (Figure 3, Zheng et al., 2020), which is characterized by the transport of proteins from the cytoplasm to the outside of the cell and is potentially involved in EET (Kanonenberg et al., 2013; Thomas et al., 2014). Recently, a repertoire of genes encoding sulfur oxidation (Figure 3, *soxYZAB*, *dsrABEFHCMKLJOPN*, *sqr*, *sorAB*, *tetH*, and *doxAD*) within the genome of *Methylovirgula thiovorans* strain HY1 suggested potential utilization of various reduced sulfur compounds for growth (Gwak et al., 2022). However, no genetic or experimental evidence of SO_4_^2−^ reduction has been found in MOB until now, perhaps because of the low thermodynamic energy yield to support life from this reaction under anoxia (Table S1, Knittel & Boetius, 2009).

## FUTURE PERSPECTIVES

Firstly, new branches of MOB have been found continuously over the past two decades (Schmitz et al., 2021), indicating that the full complement of methanotroph diversity is not yet known (Ahmadi & Lackner, 2024). Observations of MOB in anoxic aquatic ecosystems have updated the paradigm that MOB can only inhabit oxic conditions (Reis et al., 2024). Thus, it is necessary to expand investigations in global anoxic areas to unveil new members, especially the oxygen minimum zone of oceans and anoxic hypolimnion layers of deep lakes. Secondly, the source of the oxygen atom remains enigmatic when CH_4_ is transformed to CO_2_ using alternative electron acceptors by MOB independently or in combination with other microorganisms (Shi et al., 2021) (Figure 2iii,iv). Given the ancient atmosphere was characterized by limited O_2_ but abundant CH_4_ (Kasting, 1993), metabolic flexibility under anoxia besides conventional aerobic pathway maybe an important lifestyle for MOB before O_2_ was present on Earth. Therefore, clarifying the source of the oxygen atom is helpful in revealing the mechanisms of adaptation to hypoxic and anoxic environments and in understanding the metabolic strategies of MOB in ancient atmospheric circumstances. Additionally, with more MOB strains isolated from anoxic aquatic environments, the corresponding genomic information is needed to construct their synergistic evolutionary history with Earth, especially during the Great Oxygenation Event (GOE) (Lyons et al., 2014). Thirdly, although recent case studies have shown that CH_4_ oxidation mediated by MOB under anoxia significantly reduces CH_4_ emission in freshwater lakes (Li et al., 2023; Milucka et al., 2015), it is urgent to re‐evaluate the contribution of MOB to CH_4_ mitigation on a larger anoxic scale in aquatic ecosystems, which account for half of the global CH_4_ emission (Rosentreter et al., 2021). Lastly, if NO_X_^−^ are the terminal electron acceptors, Gammaproteobacterial‐MOB may lead to a net production of the far more potent greenhouse gas N_2_O (Griffis et al., 2017; Stein & Lidstrom, 2024), resulting in further climate change even though CH_4_ is consumed (Kits et al., 2015). Therefore, it is necessary to consider the net greenhouse effect of CH_4_ oxidation by MOB under O_2_‐limited conditions. Given the positive feedback between greenhouse CH_4_ emission and ubiquitous aquatic deoxygenation (Bonaglia et al., 2022), the role of MOB in anoxic environments needs to be thoroughly understood.

## AUTHOR CONTRIBUTIONS

**Biao Li:** Conceptualization; data curation; visualization; writing – original draft; writing – review and editing; funding acquisition. **Zhendu Mao:** Methodology; software; visualization. **Jingya Xue:** Methodology; visualization; software; funding acquisition. **Peng Xing:** Writing – review and editing; funding acquisition; supervision. **Qinglong L. Wu:** Supervision; conceptualization; writing – review and editing; funding acquisition.

## CONFLICT OF INTEREST STATEMENT

The authors declare that they have no known competing financial interests or personal relationships that could have appeared to influence the work reported in this paper.

## Supporting information


**Data S1.** Supporting information

## Data Availability

The data that support the findings of this study are available on request from the corresponding author. The data are not publicly available due to privacy or ethical restrictions. Supporting Materials can be accessed at Figshare and the link is http://doi.org/10.6084/m9.figshare.26755312.
