# Supplementary material for: Metabolic versatility of aerobic methane‐oxidizing bacteria under anoxia in aquatic ecosystems
Source: Environ Microbiol Rep. 2024 Sep 4;16(5):e70002. doi: 10.1111/1758-2229.70002 (PMC11374530; doi:10.1111/1758-2229.70002)
Supplement: Supplementary file 1 — Data S1. Supporting information [file EMI4-16-e70002-s001.docx]

***Supplementary Information***

**Metabolic versatility of aerobic methane-oxidizing bacteria under anoxia in aquatic ecosystems**

Biao Li ^1, 2^, Zhendu Mao ^3^, Jingya Xue ^4^, Peng Xing ^1, 2^, Qinglong L. Wu ^1, 2, 3, 5, 6, *^

^1^ Key Laboratory of Lake and Watershed Science for Water Security, Nanjing Institute of Geography and Limnology, Chinese Academy of Sciences, Nanjing 210008, China

^2^ State Key Laboratory of Lake Science and Environment, Nanjing Institute of Geography and Limnology, Chinese Academy of Sciences, Nanjing 210008, China

^3^ Center for Evolution and Conservation Biology, Southern Marine Science and Engineering Guangdong Laboratory (Guangzhou), Guangzhou 511458, China

^4^ School of Geographical Sciences, Nanjing Normal University, Nanjing 210023, China

^5^ Sino-Danish Center for Education and Research, University of Chinese Academy of Sciences, Beijing 100039, China

^6^ The Fuxianhu Station of Plateau Deep Lake Research, Chinese Academy of Sciences, Yuxi 653100, China

^*^ Corresponding author: Prof. Dr. Qinglong L. Wu ([qlwu@niglas.ac.cn](mailto:qlwu@niglas.ac.cn)).

Email address of all other co-authors: Biao Li ([libiao@niglas.ac.cn](mailto:libiao@niglas.ac.cn)), Zhendu Mao ([maozhendu@126.com](mailto:maozhendu@126.com)), Jingya Xue ([xuejingya3679@163.com](mailto:xuejingya3679@163.com)), Peng Xing ([pxing@niglas.ac.cn](mailto:pxing@niglas.ac.cn)).

**Fig. S1 Temporal distribution of publications and citations.** The gray bar and red line represent the numbers of publications and citations per year, respectively. We searched the Science Citation Index (SCI) Expanded Database, Web of Science using the topic words (aerobic methane oxidizing bacteria* or aerobic CH_4_ oxidizing bacteria* or aerobic methanotrophs*) and (anoxic water* or anoxic sediment*), and found 202 articles about MOB under anoxic aquatic ecosystems. The growth of publications and citations along year shows an increasing interest in this topic. Then, a careful check for all these literatures was taken to make sure suitable papers were selected, and publication bias was minimized using the following three criteria: (1) the detected MOB were present in aquatic ecosystems such as oceans, hydrothermal vents, lakes, and reservoirs or enriched cultures/ isolated strains from the habitats above (2) the presences of MOB were verified by direct evidences, such as lipid biomarkers, fluorescence in situ hybridization (FISH), quantitative PCR, amplicon sequencing, (meta)genome sequencing, and (meta)transcriptome sequencing; (3) the apparent anoxic conditions were direct determined by dissolved oxygen (DO) detection and inert gas purge for field samplings and laboratory systems, respectively. Finally, a total of 65 peer-reviewed publications were selected following application of the criteria above.

**

**

**Table. S1 Standard free energies (kJ/mol CH_4_) for CH_4_ oxidation coupled with different electron acceptors.**

| Reactions | ΔG^0^ | References |
| --- | --- | --- |
| CH_4_ + O_2_ → CO_2_ + 2H_2_O | -820 | James et al., 2016 |
| CH_4_ + 8/3NO_2_^-^ + 8/3H^+^ → CO_2_ + 4/3N_2_ + 10/3H_2_O | -928 | Raghoebarsing et al., 2006 |
| CH_4_ + 4NO_3_^-^ → CO_2_ + 4NO_2_^-^ + 2H_2_O | -503 | Haroon et al., 2013 |
| CH_4_ + 4MnO_2_ + 7H^+^ → HCO_3_^-^ + 4Mn^2+^ + 5H_2_O | -494 | He et al., 2018 |
| CH_4_ + 8Fe(OH)_3_ + 15H^+^ → HCO_3_^-^ + 8Fe^2+^ + 21H_2_O | -81.6 | He et al., 2018 |
| CH_4_ + SO_4_^2-^ → HCO_3_^-^ + HS^-^ + H_2_O | -16 | Boetius et al., 2000 |

**References**

Boetius, A., Ravenschlag, K., Schubert, C., Rickert, D., Widdel, F., Gieseke, A., Amann, R., Jørgensen, B., Witte, U., Pfannkuche, O., 2000. A marine microbial consortium apparently mediating anaerobic oxidation of methane. Nature. 407, 623-626.

Haroon, M., Hu, S., Shi, Y., Imelfort, M., Keller, J., Hugenholtz, P., Yuan, Z., Tyson, G., 2013. Anaerobic oxidation of methane coupled to nitrate reduction in a novel archaeal lineage. Nature. 500, 567-570.

He, Z.F., Zhang, Q.Y., Feng, Y.D., Luo, H.W., Pan, X.L., Gadd, G.M. 2018. Microbiological and environmental significance of metal-dependent anaerobic oxidation of methane. Sci Total Environ. 610-611, 759-768.

James, R.H., Bousquet, P., Bussmann, I., Haeckel, M., Kipfer, R., Leifer, I., Niemann, H., Ostrovsky, I., Piskozub, J., Rehder, G., Treude, T., Vielstädte, L., Greinert, J. 2016. Effects of climate change on methane emissions from seafloor sediments in the Arctic Ocean: A review. Limnol Oceanogr. 61, S283-S299.

Raghoebarsing, A.A, Pol, A., van de Pas-Schoonen, K.T., Smolders, A.J., Ettwig, K.F., Rijpstra, W.I., Schouten, S., Damste, J.S., Op den Camp, H.J., Jetten, M.S., Strous, M. 2006. A microbial consortium couples anaerobic methane oxidation to denitrification. Nature. 440, 918-921.
